# The Global Burden of Periodontal Disease: A Narrative Review on Unveiling Socioeconomic and Health Challenges

**DOI:** 10.3390/ijerph22040624

**Published:** 2025-04-16

**Authors:** Nada Tawfig Hashim, Rasha Babiker, Vivek Padmanabhan, Azza Tagelsir Ahmed, Nallan C. S. K. Chaitanya, Riham Mohammed, Sivan Padma Priya, Ayman Ahmed, Shadi El Bahra, Md Sofiqul Islam, Bakri Gobara Gismalla, Muhammed Mustahsen Rahman

**Affiliations:** 1 Department of Periodontics, RAK College of Dental Sciences, RAK Medical & Health Sciences University, Ras-AlKhaimah 12973, United Arab Emirates; mustahsen@rakmhsu.ac.ae; 2 Department of Physiology, RAK College of Medical Sciences, RAK Medical & Health Sciences University, Ras-AlKhaimah 11127, United Arab Emirates; rashababiker@rakmhsu.ac.ae; 3 Department of Pediatric and Preventive Dentistry, RAK College of Dental Sciences, RAK Medical & Health Sciences University, Ras-AlKhaimah 12973, United Arab Emirates; vivek.padmanabhan@rakmhsu.ac.ae; 4 Department of Pediatric Dentistry, University of Illinois Chicago, Chicago, IL 60607, USA; azzahmed@uic.edu; 5 Department of Oral Medicine and Radiology, RAK College of Dental Sciences, RAK Medical & Health Sciences University, Ras-AlKhaimah 12973, United Arab Emirates; krishna.chytanya@rakmhsu.ac.ae; 6 Department Oral Surgery, RAK College of Dental Sciences, RAK Medical & Health Sciences University, Ras-AlKhaimah 12973, United Arab Emirates; riham.abdelraouf@rakmhsu.ac.ae; 7 Department of Oral Pathology, RAK College of Dental Sciences, RAK Medical & Health Sciences University, Ras-AlKhaimah 12973, United Arab Emirates; sivan.padma@rakmhsu.ac.ae; 8 Department of Periodontology and Implantology, Nile University, Khartoum 11115, Sudan; ayman.ahmed@nileuniversity.edu.sd; 9 Department of Prosthodontics, RAK College of Dental Sciences, RAK Medical & Health Sciences University, Ras-AlKhaimah 12973, United Arab Emirates; shadi.elbahra@rakmhsu.ac.ae; 10 Department of Operative Dentistry, RAK College of Dental Sciences, RAK Medical & Health Sciences University, Ras-AlKhaimah 12973, United Arab Emirates; sofiqul.islam@rakmhsu.ac.ae; 11 Department of Oral Rehabilitation, Faculty of Dentistry, University of Khartoum, Khartoum 11115, Sudan; bakri.gobara@uofk.edu

**Keywords:** periodontal disease, global health, socioeconomic impact, systemic inflammation, cardiovascular disease, diabetes mellitus, respiratory infections, adverse pregnancy outcomes, quality of life, public health strategies, oral health integration

## Abstract

Periodontal disease is a prevalent chronic inflammatory condition that impacts over a billion people worldwide, leading to substantial tooth loss, reduced quality of life, and heightened systemic health risks. This narrative review synthesizes current evidence regarding the global burden of periodontal disease, its established associations with systemic conditions including cardiovascular disease, diabetes, adverse pregnancy outcomes, respiratory infections, and neurodegenerative disorders, and its significant socioeconomic implications. The review focused on the following research question: What is the global burden of periodontal disease, and how do its systemic and socioeconomic implications necessitate integrated public health strategies? A structured search of the PubMed, Scopus, and WHO databases from 2000 to 2024 was conducted to identify relevant literature using key terms, including “periodontal disease”, “global burden”, “systemic inflammation”, and “public health strategies”. Out of 312 initially identified articles, 175 satisfied the inclusion criteria for the final synthesis. The findings underscore the significance of periodontal disease as a modifiable risk factor for various noncommunicable diseases, the influence of healthcare disparities on disease progression, and the critical necessity for integrated public health strategies to mitigate the global burden of periodontal disease and its consequences. The review concludes that coordinated policy reform, health system integration, and enhanced research efforts are crucial for mitigating the global burden of periodontal disease and advancing health equity.

## 1. Introduction

Periodontal diseases include a range of clinical conditions where initiating an inflammatory response leads to the deterioration of the structures that hold the teeth in place, including the loss of supporting bone [1]. Periodontal disease is a prevalent condition affecting the oral cavity, and if left untreated, it is the primary reason for tooth loss in adults [2]. Historically, dental plaque, microbial byproducts, and the host immunological response have been the leading causes of periodontal diseases [3]. While recent research has shown that environmental, behavioral, and genetic variables may contribute to the advancement of periodontal disease, it is crucial to recognize that all forms of periodontitis are infectious [4,5,6].

Bacteria are the leading cause of periodontal disorders, although new data suggest that yeast and herpes viruses may also play a role as potential pathogens [7]. However, our understanding of the disease-causing mechanism has been hindered because it often stems from a polymicrobial infection, including native species with limited ability to cause disease [8]. Gingival inflammation without bone loss is a sign of the disease’s reversible phase, known as gingivitis [9]. Conversely, periodontitis, a more severe condition, worsens the alveolar bone and may ultimately lead to tooth loss [10].

Recent advances in periodontal microbiology have moved beyond the classical “red complex” paradigm toward the more comprehensive polymicrobial synergy and dysbiosis (PSD) model. According to this model, periodontal disease is not caused by a single pathogen but rather by a synergistic microbial community that disrupts host–microbe homeostasis [11]. Keystone pathogens such as *Porphyromonas gingivalis* (*P. gingivalis*) can manipulate the host immune response, leading to an inflammatory environment that supports the growth of pathobionts and drives tissue destruction. This concept of dysbiosis, where microbial imbalance contributes to disease progression, provides a more accurate framework for understanding the etiopathogenesis of periodontitis. It also highlights the importance of considering the host response and microbial interactions in both the prevention and management of the disease [11].

Periodontitis can be classified into stages I, II, III, and IV based on the degree of bone loss and the severity of the condition. Most patients experience stage I and II periodontitis, which are subtle diseases characterized by damage associated with bacterial plaque and calculus [12]. These stages occur due to a polymicrobial infection with varying microbial patterns. In contrast, stages III and IV periodontitis are defined by rapid loss of attachment and bone destruction, often disproportionate to the amount of microbial deposits present [13].

The Molar Incisor form of periodontitis is a distinct disease compared to other forms of periodontitis. It typically occurs during adolescence, when the incidence of periodontal disease is usually low [14]. The subgingival microbiota in this periodontitis form strongly associates (96.5%) with a specific bacterium called *Aggregatibacter actinomycetemcomitans* (*A. actinomycetemcomitans*). Bone resorption in this disease progresses three to four times faster than in permanent periodontitis. It may also spontaneously stop and is localized to specific teeth, namely the first molars and incisors. Additionally, this disease tends to cluster in families, suggesting a genetic predisposition to the disease [15].

In 2021, the 74th World Health Assembly of the World Health Organization (WHO) recognized oral health as a vital component of overall well-being and adopted a resolution emphasizing the necessity of disease prevention and control in this area [16].

Oral diseases continue to present a significant global health challenge, affecting a large number of individuals and imposing a considerable economic burden on societies. These conditions not only worsen health inequities by disproportionately impacting disadvantaged and socially vulnerable populations but are also closely associated with noncommunicable diseases (NCDs) [17]. Alarmingly, even in high-income countries, the burden of oral diseases persists as a pressing issue, leading to decreased productivity and quality of life. Given that many oral diseases are largely preventable, the WHO has set an ambitious goal to improve global oral health by 2030. This goal aligns with its broader mission to achieve universal health coverage—ensuring equitable access to essential healthcare services without financial burden—while also addressing the increasing prevalence of NCDs [18].

**Figure 1:**
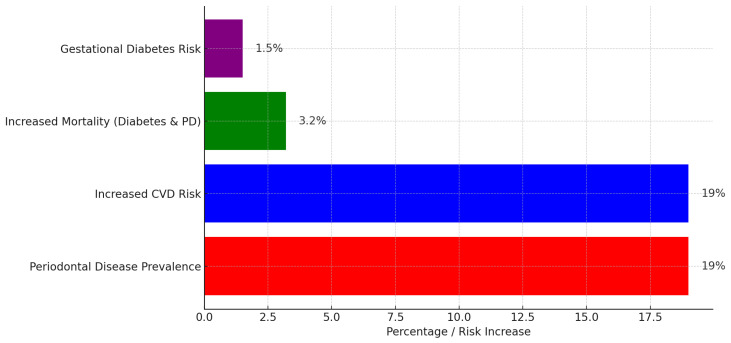
Figure illustrating the global burden of periodontal disease and its associated systemic risks. Periodontal disease prevalence is 19%, with an equal increase in cardiovascular disease (CVD) risk. Additionally, individuals with both diabetes and periodontal disease face a 3.2% rise in mortality risk, while gestational diabetes risk increases by 1.5%. The chart highlights the significant health implications of periodontal disease beyond oral health.

**Figure 2:**
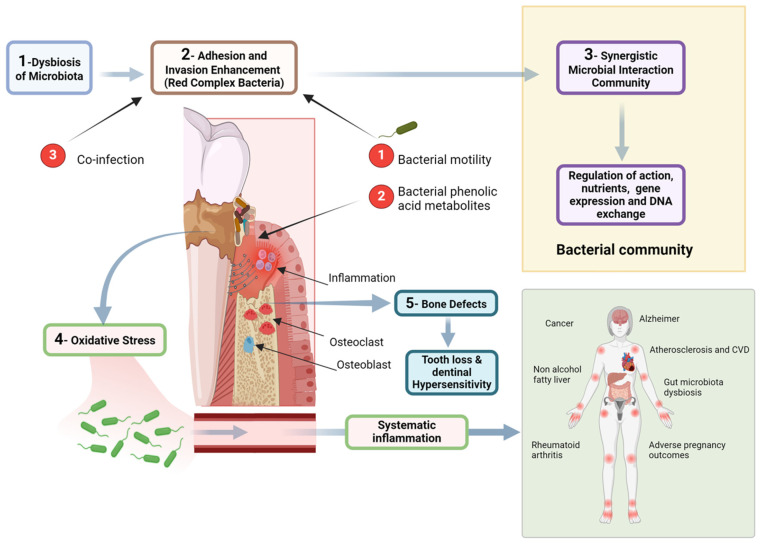
Figure illustrating the pathogenic mechanisms linking dysbiosis of the oral microbiota to systemic diseases. The process begins with microbial imbalance, leading to the adhesion and invasion of red complex bacteria, which enhance pathogenicity through motility and phenolic acid metabolites. A synergistic bacterial community further regulates gene expression and nutrient exchange, promoting inflammation and oxidative stress. These factors contribute to bone defects, tooth loss, and dentin hypersensitivity. Additionally, systemic inflammation resulting from periodontal disease is associated with broader health implications, including cardiovascular disease, Alzheimer’s disease, rheumatoid arthritis, cancer, and adverse pregnancy outcomes. The diagram highlights the intricate relationship between oral and systemic health, emphasizing the need for periodontal disease management in preventing systemic conditions. Created with BioRender.com (accessed on 5 March 2025).

Periodontal disease has been increasingly recognized among these conditions for its association with systemic health complications [2]. In developed countries, where aging populations retain more of their natural teeth, the incidence of periodontal disease is rising [19]. This condition is not only a primary cause of tooth loss but also a well-established risk factor for several major NCDs, including cardiovascular disease, diabetes, respiratory disorders, pregnancy-related complications, obesity, cancer, and neurodegenerative conditions such as Alzheimer’s disease [20,21,22,23,24,25] (Figure 1 and Figure 2).

## 2. Methodological Approach and Literature Synthesis Strategy

This narrative review focuses on the global prevalence of periodontal disease and its systemic and socioeconomic implications, hence advocating for the incorporation of public health policies. The main aim of this study is to compile existing data about the global prevalence of periodontal disease and its established correlations with cardiovascular disease, diabetes, adverse pregnancy outcomes, respiratory infections, and economic consequences. The study employed a thematic synthesis approach to elucidate the role of periodontal disease as a modifiable risk factor for many noncommunicable diseases while simultaneously emphasizing its position as a neglected global health policy concern for immediate attention. A thorough search was performed by utilizing the PubMed, Scopus, and World Health Organization (WHO) databases to find relevant literature, with an emphasis on works published between January 2000 and March 2024. The search included combinations of terms like “periodontal disease”, “global burden”, “systemic inflammation”, “oral-systemic health”, and “public health strategies”. A preliminary collection of 312 items was obtained. Following the elimination of duplicates and the assessment of titles and abstracts for topic pertinence, 274 articles were chosen for comprehensive examination. Studies were considered if they examined the epidemiology, systemic relationships, or public health ramifications of periodontal disease and were published in peer-reviewed publications. The exclusion criteria included case reports, editorials, opinions, and papers that focused only on local solutions without larger structural or policy significance. In conclusion, 175 articles were included in the final synthesis. 

Although no formal quality appraisal tool was used, consistent with the narrative review format, each article was selected based on its methodological soundness, citation strength, and alignment with the thematic focus of the review. The comprehensive literature demonstrates a multidisciplinary synthesis of clinical, epidemiological, and policy-oriented viewpoints, underscoring the need for prompt, cohesive public health interventions to alleviate the escalating impact of periodontal disease on individual and community health.

## 3. Global Prevalence of Periodontal Disease

Periodontal disease, a condition of persistent concern, continues to be a prevalent issue worldwide. The Global Burden of Disease (GBD) Study 2019 reported approximately 1.1 billion cases of severe periodontitis in 2019, a number that has nearly doubled since 1990. The consistent upward trend in both age-standardized incidence and prevalence rates highlights the need for continuous research and intervention [26].

**Figure 3:**
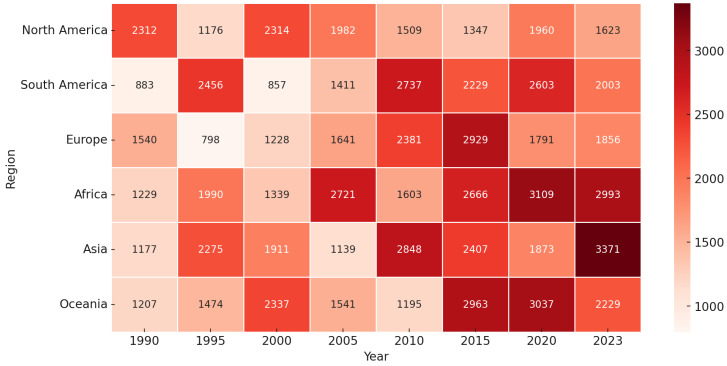
Figure displaying the progression of a variable (e.g., periodontal disease prevalence or healthcare burden) across different regions from 1990 to 2023. Each cell represents the value for a specific region and year, with darker shades indicating higher values. The color gradient ranges from light (lower values) to deep red (higher values), signifying the increasing trend over time. Notably, Africa and Asia exhibit a significant rise in values, particularly in the later years, suggesting a growing burden in these regions. This visualization highlights regional disparities and temporal trends, emphasizing the need for targeted interventions.

The burden of periodontitis varies geographically. In 2019, Western Sub-Saharan Africa was identified as a high-risk region, with the Gambia experiencing the highest burden. Globally, periodontitis ranks as the 11th most prevalent disease [26] (Figure 3).

Its occurrence is notably higher in low- and middle-income countries due to limited access to dental care, inadequate oral hygiene practices, and a lack of oral health education [27]. However, despite better access to dental services in high-income countries, periodontitis remains a significant public health concern, largely due to contributing factors such as smoking, diabetes, and an aging population [28]. Table 1.

## 4. Socioeconomic Impact

The socioeconomic impact of periodontal disease is profound, carrying significant implications for both public health and economic productivity. The global burden of periodontitis accounts for an estimated 3.5 million years lived with disability and results in an annual loss of USD 54 billion in productivity. The total cost of oral diseases, of which periodontitis is a major component, reaches approximately USD 442 billion each year [29]. This burden is anticipated to grow due to demographic changes, including the aging population and improved tooth retention, which extend exposure to risk factors [18]. The disease disproportionately impacts vulnerable populations, worsening social inequalities, as access to professional periodontal care remains limited in many areas. Moreover, untreated periodontitis contributes to systemic health issues, such as cardiovascular disease and diabetes, further increasing healthcare costs [30].

The financial burden of periodontal disease is substantial. Direct costs encompass expenses associated with dental treatments, including professional cleanings, periodontal surgeries, and maintenance therapies [31]. Indirect costs involve lost productivity due to absenteeism, presenteeism, and premature tooth loss, which can affect an individual’s capacity to work and overall quality of life. Periodontal disease disproportionately impacts disadvantaged populations, contributing to health disparities. Poor oral health can result in social stigmatization, affecting mental health and employability, thus further entrenching poverty [32,33]. Table 2.

Addressing periodontal disease through preventive measures, early diagnosis, and improved public awareness is essential to mitigating its widespread economic and health consequences. Implementing evidence-based interventions, such as professional periodontal screening and self-care education, aligns with global public health priorities and could significantly reduce both the human and economic toll of this pervasive disease [31].

## 5. Impact of Periodontal Disease on Immune Response and Systemic Health

**Figure 4:**
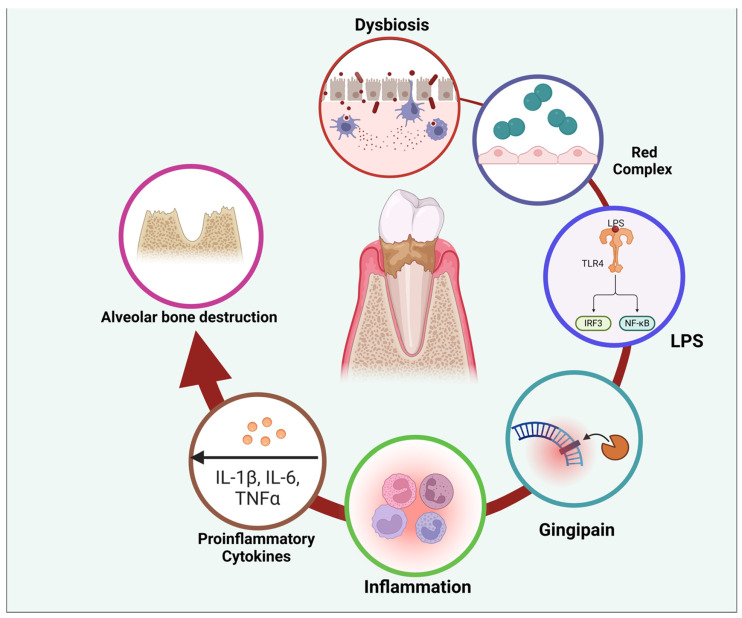
Figure illustrating the pathogenic cycle of periodontal disease, beginning with dysbiosis—an imbalance in the oral microbiota—leading to the dominance of red complex bacteria. These bacteria produce virulence factors such as lipopolysaccharides (LPS) and gingipains, which trigger an immune response. LPS activates Toll-like receptors (TLRs), stimulating intracellular signaling pathways (IRF3 and NF-κB) that promote the release of proinflammatory cytokines, including IL-1β, IL-6, and TNFα. This inflammatory cascade contributes to tissue damage and alveolar bone destruction, perpetuating disease progression. The cycle highlights the interplay between microbial factors and host immune responses in periodontal disease pathogenesis. Created with BioRender.com (accessed on 5 March 2025).

Periodontal disease results from a complex relationship between microorganisms and the host’s immune response [34]. The main factor is dental plaque, a microbial biofilm that triggers the inflammatory reaction. Notable pathogens include *Porphyromonas gingivalis*, *Tannerella forsythia*, and *Treponema denticola* [35]. The host’s immunological response to these infections, which includes both innate and adaptive immunity, results in the secretion of inflammatory substances such as cytokines, chemokines, and matrix metalloproteinases. This reaction leads to the degradation of periodontal tissues, such as connective tissue and alveolar bone [36] (Figure 4).

Periodontal disease begins when the normal equilibrium of microorganisms in the mouth is disrupted, leading to changes at the molecular and physiological levels [20].

Particular microorganisms in the oral cavity trigger immunological responses, activating the innate immune cells (macrophages, dendritic cells, natural killer cells, monocytes, and neutrophils) and the adaptive immune cells (B and T lymphocytes) [37,38].

This results in the production and release of inflammatory agents, including interferon-γ, interleukin-17, tumor necrosis factor, and other interleukins. Additionally, enzymes like matrix metalloproteinases are secreted, leading to the breakdown of collagen. The occurrence of inflammation in periodontal disease serves as a protective response against bacterial infections that may infiltrate deeper tissues, including bones [21].

Nevertheless, if this ongoing inflammation is not well controlled, it may cause irreversible harm to the tissues around the teeth, leading to periodontitis. Periodontitis is manifested by periodontal pockets, attachment loss, gingival recession, tooth mobility, tooth migration, and eventually, tooth loss. The damaged area becomes a pivotal location where localized inflammation may significantly influence overall health [20].

The oral cavity has various microorganisms, ranging from 500 to 700 species, collectively known as the oral microbiota. These microbes may be found in saliva, gingival epithelium, and dental plaque [39]. The lipopolysaccharide produced by *Porphyromonas gingivalis* induces persistent inflammation in the gingiva and is associated with systemic inflammatory disorders [40] (Figure 4).

The IL-17 signaling pathway is essential for regulating immunological responses and chronic inflammation, which may disrupt the balance of microbial populations [41].

IL-17 may provide a favorable habitat for pathogenic microorganisms, exacerbating periodontal inflammation. IL-23-dependent IL-17 signaling has been linked to increased bacterial proliferation in periodontal disease. Nevertheless, inhibiting the IL-17 cascade might halt the abnormal proliferation of bacteria [42].

Endogenous inflammatory mediators released during periodontitis can enter the bloodstream, potentially influencing distant organs and contributing to systemic inflammatory dysregulation. This mechanism may explain the broader implications of periodontitis on systemic inflammation. Individuals with periodontitis often show elevated leukocyte counts and heightened levels of systemic inflammatory markers, such as C-reactive protein. This evidence underscores the bidirectional relationship between localized periodontal inflammation and overall health. Moreover, research has explored the link between oxidative stress and systemic inflammation in severe periodontal disease, further highlighting its role in systemic disease progression [21].

**Figure 5:**
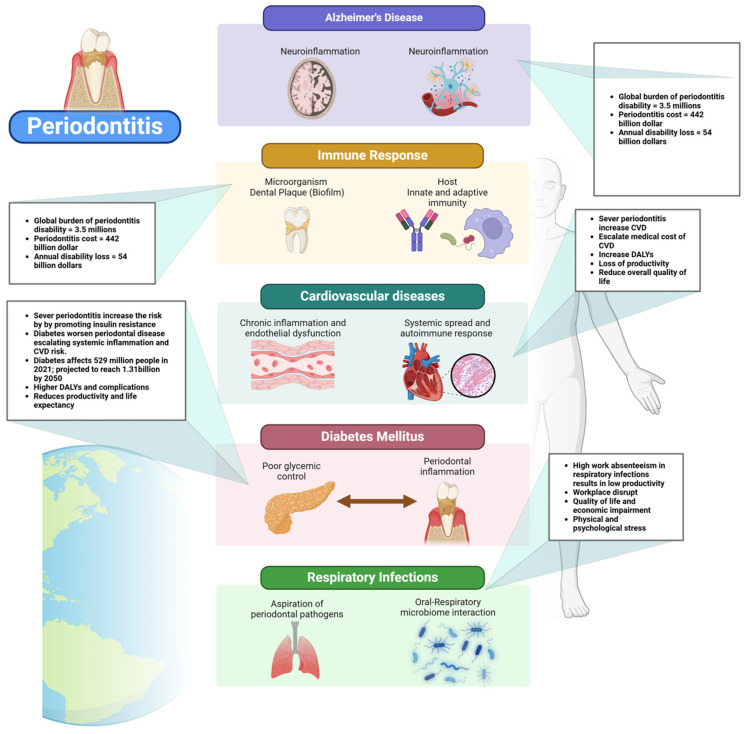
Figure illustrating the systemic impact of periodontitis, linking it to various health conditions. Periodontitis, characterized by chronic inflammation and bacterial dysbiosis, triggers an immune response involving microbial biofilms, virulence factors, and host defense mechanisms. The inflammatory cascade contributes to several systemic diseases, including Alzheimer’s disease (via neuroinflammation), cardiovascular diseases (through systemic spread of inflammatory mediators and endothelial dysfunction), diabetes mellitus (by exacerbating poor glycemic control and increasing inflammation), and respiratory infections (due to the aspiration of periodontal pathogens). The diagram also highlights the global burden of periodontitis, emphasizing its prevalence and association with increased disease risks, disability-adjusted life years (DALYs), and higher morbidity rates in individuals over 54 years of age. Created with BioRender.com (accessed on 5 March 2025).

Cardiac infarction and periodontal disease are associated with oxidative stress and inflammatory processes [43]. As periodontal disease develops, the expression of antioxidant enzymes, like SOD2, is increased due to ongoing inflammation [44] (Figure 5).

Chronic conditions, including diabetes, obesity, cardiovascular illnesses, and neurological diseases, often display a moderate state of inflammation, which has been associated with periodontal disease. The exact causal relationship between periodontal inflammation and chronic systemic illnesses is still debated. Levels of inflammation within the range of 3 mg/L to 10 mg/L of C-reactive protein have been shown to signify low-grade inflammation [21,45].

Empirical evidence substantiates the connection between periodontal disease and systemic inflammation; however, a precise cause-and-effect relationship has not been conclusively established [22,45,46].

Multiple studies have shown that periodontal treatment decreases the prevalence of systemic inflammatory markers and potentially decreases the risk of cardiovascular disease [47]. Periodontal treatment is also associated with modest improvements in glycated hemoglobin levels in persons with type 2 diabetes; however, more comprehensive research is necessary [23].

Although there is no definitive evidence of particular benefits for obstetric difficulties, the use of preventative therapies in women with periodontitis is commonly considered to be a prudent strategy [24].

The complex interconnection between periodontal disease, the immune response, and overall health emphasizes the need for comprehensive approaches to control and prevent periodontitis to reduce the impact of its wider health consequences.

## 6. Cardiovascular Disease

Cardiovascular diseases (CVDs) are inflammatory diseases of coronary arteries accompanying atheroma formation that can spawn impairment and, in severe cases, death. CVDs are the leading cause of death around the world [48].

Periodontal disease has been extensively linked to cardiovascular disease (CVD). Chronic inflammation from periodontitis can lead to endothelial dysfunction and atherosclerosis [49].

Periodontal pathogens such as *Porphyromonas gingivalis* and *Aggregatibacter actinomycetemcomitans* can enter the bloodstream, promoting the formation of atheromatous plaques [50]. Studies have shown that individuals with periodontitis have a significantly higher risk of myocardial infarction and stroke [51,52]. Inflammatory mediators, such as C-reactive protein and interleukin-6, play a crucial role in the pathogenesis of CVD in periodontitis patients.

### 6.1. The Pathophysiological Link Between Periodontitis and Cardiovascular Diseases

#### 6.1.1. Chronic Inflammation and Endothelial Dysfunction

Periodontitis causes chronic inflammation due to persistent bacterial infection. This inflammation is characterized by elevated levels of systemic inflammatory markers, such as C-reactive protein (CRP), interleukin-6 (IL-6), and tumor necrosis factor-alpha (TNF-α) [20].

These inflammatory mediators contribute to endothelial dysfunction, a critical early step in the development of atherosclerosis. Endothelial cells line the interior surface of blood vessels and play a vital role in vascular homeostasis. Inflammation impairs the production of nitric oxide, a vasodilator, leading to reduced vessel elasticity and increased vascular resistance [53].

Chronic inflammation promotes the accumulation of low-density lipoproteins (LDL) in the arterial walls, leading to the formation of atherosclerotic plaques. The presence of these plaques narrows the arteries and restricts blood flow, increasing the risk of cardiovascular events such as myocardial infarction (heart attack) and stroke [54].

#### 6.1.2. Bacterial Invasion and Systemic Spread

Periodontal pathogens, including *Porphyromonas gingivalis*, *Aggregatibacter actinomycetemcomitans*, and *Treponema denticola*, can enter the bloodstream during routine activities like chewing and tooth brushing in individuals with periodontitis. This bacteremia facilitates the systemic spread of these pathogens [55].

Once in the bloodstream, periodontal bacteria can adhere to and invade the endothelial cells of blood vessels. They can be directly involved in the formation and progression of atherosclerotic plaques by stimulating the inflammatory response and increasing the deposition of lipids in the arterial walls [56].

The immune system responds to these bacterial invaders by releasing additional inflammatory mediators and recruiting immune cells to the site of infection, further contributing to endothelial damage and plaque instability [57].

#### 6.1.3. Molecular Mimicry and Autoimmunity

Some periodontal pathogens possess antigens that mimic host molecules. This molecular mimicry can trigger an autoimmune response, where the immune system mistakenly attacks its tissues, including the endothelial cells lining the blood vessels [58,59].

*Porphyromonas gingivalis* is a prominent periodontal pathogen that exhibits molecular mimicry, which plays a significant role in chronic periodontitis and atherosclerosis progression [60].

This bacterium produces a heat shock protein, specifically HSP60, closely resembling the human version of HSP60, a protein found in the endothelial cells lining blood vessels. In its effort to target and eliminate the bacterial HSP60, the immune system can mistakenly recognize the host’s HSP60 as foreign due to their structural similarity [61,62].

This mistaken identity triggers an immune response against the endothelial cells, leading to inflammation and contributing to the development and progression of atherosclerosis. This example underscores the dual impact of *Porphyromonas gingivalis* in oral and cardiovascular health, illustrating the complex interplay between periodontal infection and systemic inflammatory diseases [63].

### 6.2. The Broader Impact of Periodontal Disease on Cardiovascular Health and Society

#### 6.2.1. Increased Cardiovascular Morbidity and Mortality

Periodontal disease is increasingly recognized as a significant contributor to the global burden of cardiovascular disease (CVD), with growing evidence linking periodontitis to elevated risks of myocardial infarction and stroke [51]. The Global Burden of Disease (GBD) study highlights that severe periodontitis affects over a billion people worldwide, and its systemic impact extends beyond oral health, contributing to increased morbidity and mortality from CVD [64]. This growing prevalence not only places an immense strain on healthcare systems but also escalates direct medical costs associated with emergency care, hospitalizations, surgical interventions, and long-term pharmacological treatments for cardiovascular complications [64]. The societal impact is substantial, with increased disability-adjusted life years (DALYs) and lost productivity due to chronic disease progression [64] (Figure 5). This underscores the need for a more integrated healthcare approach, one that prioritizes oral health as a fundamental component of cardiovascular disease prevention, thereby mitigating the widespread and costly impact on global health.

#### 6.2.2. Socioeconomic Consequences

Individuals suffering from periodontal disease and its cardiovascular complications often face decreased productivity due to illness, disability, and absenteeism, which impacts both their livelihoods and the broader economy [2].

The indirect costs associated with this loss of productivity, when combined with the direct healthcare costs required for managing these chronic conditions, create a significant economic burden on families and communities. This financial strain is particularly pronounced in low- and middle-income regions, where existing socioeconomic disparities can be further exacerbated, deepening the cycle of poverty and health inequality [30].

#### 6.2.3. Quality of Life and Mental Health

Chronic conditions such as periodontitis and cardiovascular disease (CVD) significantly impact physical well-being, limiting individuals’ ability to engage in daily activities and thereby reducing their overall quality of life [51,65].

The burden of managing these chronic diseases, coupled with the constant concern over the potential for acute cardiovascular events, can also lead to substantial psychological stress. This ongoing stress and anxiety often result in mental health issues, including depression and anxiety disorders, further compounding the challenges faced by those affected and diminishing their overall well-being [66].

### 6.3. Public Health and Preventive Measures

Raising awareness about the link between periodontal disease and cardiovascular health is essential for public health. Public health campaigns can play a vital role in educating communities about the importance of oral hygiene and regular dental check-ups as preventive measures. Additionally, developing integrated healthcare models that incorporate routine periodontal assessments within cardiovascular risk management programs can aid in the early detection and treatment of periodontal disease, thereby reducing the risk of cardiovascular complications. Policymakers also need to recognize the interconnectedness of oral and systemic health by promoting policies that increase access to comprehensive dental care, especially for high-risk populations, to mitigate the impact of periodontal disease on cardiovascular health [30,66].

## 7. Diabetes Mellitus

The relationship between periodontal disease and diabetes mellitus is bidirectional. Poor glycemic control in diabetic patients exacerbates periodontal inflammation, while periodontitis can impair insulin sensitivity and glycemic control, complicating diabetes management [67].

Effective periodontal treatment has been shown to improve glycemic control in diabetic patients [68]. Chronic inflammation and oxidative stress are pivotal in the interplay between periodontitis and diabetes, with inflammatory cytokines such as tumor necrosis factor-alpha (TNF-α) and interleukin-1 beta (IL-1β) playing central roles in this connection [69].

**Figure 6:**
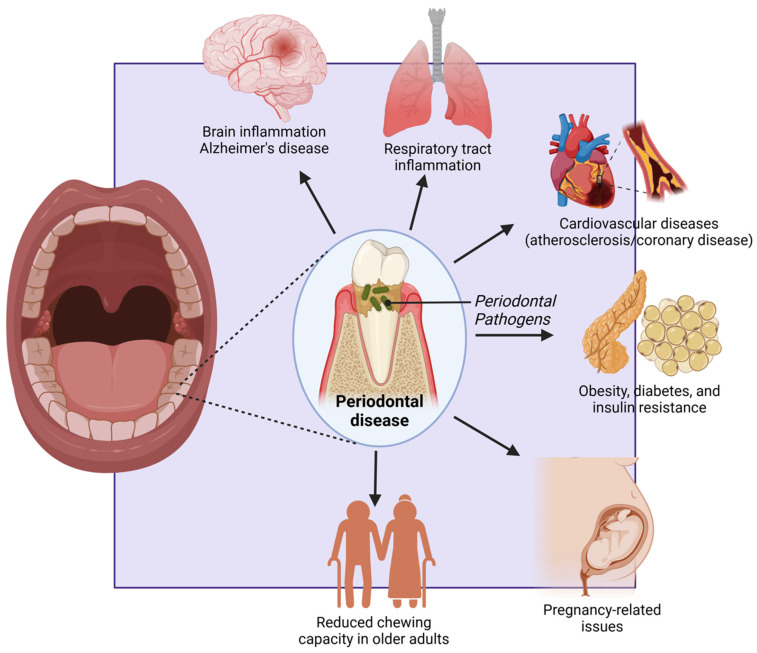
Figure highlighting the systemic effects of periodontal disease. Periodontal pathogens contribute to brain inflammation (Alzheimer’s disease), respiratory tract infections, and cardiovascular diseases (atherosclerosis/coronary disease). They are also linked to metabolic disorders like obesity, diabetes, and insulin resistance, as well as pregnancy-related complications. In older adults, periodontal disease reduces chewing capacity, affecting nutrition and overall health. Created with BioRender.com (accessed on 5 March 2025).

TNF-α, produced by macrophages in response to infections and tissue damage, is upregulated in inflamed gingival tissues during periodontitis, contributing to tissue degradation and enhancing the inflammatory response. In diabetes, TNF-α interferes with insulin signaling pathways, leading to insulin resistance and elevated blood glucose levels [70]. Similarly, IL-1β, released by immune cells in response to bacterial infection in periodontitis, contributes to the breakdown of the periodontal ligament and alveolar bone, while in diabetes, it is implicated in beta-cell dysfunction and insulin resistance [70]. The chronic inflammation driven by these cytokines in periodontitis can exacerbate systemic inflammation in diabetic patients, worsening glycemic control and creating a vicious cycle in which periodontal inflammation and metabolic dysregulation perpetuate each other [71] (Figure 6).

Understanding the pathophysiology of these conditions underscores the importance of integrated care approaches that address both oral and systemic health, particularly in patients with diabetes.

### 7.1. The Pathophysiological Link Between Periodontitis and Diabetes Mellitus

#### 7.1.1. Bidirectional Relationship

There exists a bidirectional relationship between periodontal disease and diabetes, whereby each condition can exacerbate the other. On one hand, periodontal disease triggers the release of inflammatory cytokines, such as interleukin-6 (IL-6) and tumor necrosis factor-alpha (TNF-α), which impair insulin signaling pathways, resulting in increased insulin resistance and poor glycemic control in diabetic patients [20]. Additionally, the chronic inflammation associated with periodontitis induces oxidative stress, further disrupting insulin action and contributing to hyperglycemia [72]. On the other hand, diabetes, particularly when poorly controlled, creates a favorable environment for the proliferation of periodontal pathogens, leading to more severe periodontal tissue destruction. Moreover, hyperglycemia compromises the immune response, diminishing the host’s capacity to combat infections, including those affecting the periodontium, thereby accelerating disease severity and progression [73].

#### 7.1.2. Advanced Glycation End Products (AGEs)

In diabetes, persistent hyperglycemia leads to the excessive presence of glucose in the bloodstream, which interacts with proteins through a process known as non-enzymatic glycation, resulting in the formation of advanced glycation end products (AGEs) [74].

The initial reaction involves the formation of a Schiff base between glucose and the amino groups of proteins, which over time rearranges into more stable structures, ultimately leading to AGEs. These AGEs can accumulate in tissues such as the periodontal tissues, where they alter the structure and function of collagen and other proteins [75].

In periodontitis, this accumulation exacerbates the inflammatory response, as AGEs can bind to receptors known as RAGE (Receptor for Advanced Glycation End products) on cells within the gingiva [76].

This binding triggers a cascade of inflammatory signals, increasing the production of pro-inflammatory cytokines and oxidative stress, which further damages periodontal tissues. Thus, the formation and accumulation of AGEs in the context of diabetes not only contributes to tissue damage in periodontitis but also perpetuates a cycle of chronic inflammation that exacerbates both conditions [77].

#### 7.1.3. Microvascular Complications

Diabetes-associated microvascular complications, such as those affecting the small blood vessels, can lead to significantly impaired blood flow to the periodontal tissues. This reduction in blood supply results from the thickening of capillary walls and the narrowing of the vessel lumen due to the accumulation of advanced glycation end products (AGEs) and other changes in the vascular structure. The diminished blood flow reduces the delivery of essential oxygen and nutrients to the periodontal tissues, which are critical for maintaining tissue health and supporting the body’s natural repair processes [78].

When blood flow is compromised, the ability of the periodontal tissues to recover from injury or infection is severely hindered. This impaired healing capacity makes the tissues more susceptible to damage from bacterial infections that cause periodontitis. Additionally, the reduced blood supply limits the effectiveness of the immune response in the periodontal region. Immune cells, which rely on a robust blood supply to reach areas of infection, are less able to combat bacterial invasion effectively. Consequently, the persistent presence of bacteria in the periodontal pockets leads to continuous inflammation [79]. This chronic inflammation, in turn, exacerbates the breakdown of the periodontal tissues. Inflammatory mediators such as cytokines and matrix metalloproteinases (MMPs) are produced in response to bacterial infection and are involved in the destruction of the connective tissue and bone that support the teeth [80]. The combination of impaired blood flow and chronic inflammation accelerates this destructive process, leading to the progressive loss of periodontal attachment and bone, which can result in tooth loss [81].

Furthermore, the impaired blood flow also contributes to the development of more severe forms of periodontitis. As the periodontal tissues continue to degrade, pockets form between the gingival tissues and teeth, harboring more bacteria and creating an environment conducive to further infection and inflammation. This cycle of reduced blood flow, inadequate immune response, and ongoing tissue destruction underscores the complex and interrelated mechanisms by which diabetes exacerbates periodontal disease, leading to significant oral health complications [8,82].

#### 7.1.4. Dysbiosis of the Oral Microbiota

In individuals with diabetes, the chronic hyperglycemic state can significantly alter the composition of the oral microbiota, leading to a condition known as dysbiosis, which refers to an imbalance between beneficial and pathogenic microbial communities, with a shift toward a more pathogenic microbiome [83].

This altered microbial environment plays a crucial role in the initiation and progression of periodontal disease. In diabetic patients, the oral microbiota tends to favor the overgrowth of pathogenic bacteria, particularly Gram-negative anaerobes, which are known to be more virulent [84]. Notably, species such as *Porphyromonas gingivalis*, *Tannerella forsythia*, and *Treponema denticola*, which are part of the “red complex”, are commonly found in higher concentrations in the periodontal pockets of diabetic individuals [85]. These bacteria are strongly associated with periodontitis because they can evade the host immune response, produce virulence factors such as proteases, and promote tissue destruction. Other bacteria like *Prevotella intermedia* and *Fusobacterium nucleatum* are also prevalent in the oral microbiota of diabetic patients, contributing to the inflammatory environment that characterizes periodontal disease [37].

The increase in these pathogenic bacteria is often accompanied by a decrease in beneficial commensal bacteria, such as *Streptococcus* species, which play a role in maintaining oral health by producing substances that inhibit the growth of pathogens and contribute to the overall stability of the oral microbiome. The altered microbial environment in diabetic patients does more than just increase the number of pathogenic bacteria; it also dysregulates the host immune response [86].

Hyperglycemia can impair neutrophil function, reduce chemotaxis, and decrease the overall antimicrobial capacity of the immune cells. As a result, the body’s ability to control and eliminate pathogenic bacteria is compromised, allowing these bacteria to persist and thrive. This persistent bacterial presence stimulates the continuous production of inflammatory mediators such as cytokines, including interleukin-6 (IL-6) and tumor necrosis factor-alpha (TNF-α) [87].

These cytokines further exacerbate the inflammatory response, leading to increased tissue destruction in the periodontium. The ongoing inflammation and tissue breakdown create a favorable environment for the pathogenic bacteria to continue proliferating, establishing a vicious cycle of infection and inflammation that perpetuates and worsens periodontal disease in individuals with diabetes [88]. This dysbiotic state not only accelerates the progression of periodontal disease but also makes it more challenging to manage, requiring more aggressive and comprehensive treatment strategies [89]. Understanding the specific shifts in oral microbiota and the associated immune dysregulation in diabetic patients is crucial for developing targeted therapies that address both the microbial and inflammatory aspects of periodontal disease in this population.

### 7.2. The Broader Impact of Periodontal Disease and Diabetes on Society

#### 7.2.1. Increased Healthcare Costs

The bidirectional relationship between periodontal disease and diabetes significantly increases the complexity of managing both conditions, leading to higher healthcare costs. The financial burden of periodontal disease and diabetes is particularly significant in communities where both conditions are highly prevalent, placing a strain on families and healthcare systems [72,90].

The global economic burden of diabetes is projected to rise substantially, posing serious socioeconomic challenges. By 2030, diabetes-related costs are estimated to reach up to $2.5 trillion, representing approximately 2.2% of global GDP [91]. Even if global health targets are met, the financial impact of diabetes will not decline significantly, highlighting the need for urgent policy interventions (Figure 5). The economic burden includes both direct medical expenses and indirect costs such as lost productivity due to absenteeism, presenteeism, and premature mortality. The disease disproportionately affects middle-income countries, where healthcare infrastructure is often less equipped to handle the growing prevalence of diabetes [92]. North America and East Asia are projected to bear the highest financial burden, while Latin America is expected to experience the largest cost increase relative to GDP. The rising economic strain calls for improved prevention strategies, early diagnosis, and better disease management to mitigate the financial and societal impacts of diabetes [91].

#### 7.2.2. Productivity Loss

Individuals suffering from severe periodontal disease and poorly controlled diabetes are more likely to miss work due to illness, dental procedures, and complications, leading to increased absenteeism that affects both individual productivity and economic stability [93].

Chronic health issues can also result in reduced workforce participation, further impacting community economic growth and development, as a significant portion of the population may be unable to contribute effectively to the workforce due to these debilitating conditions [94].

#### 7.2.3. Quality of Life and Mental Health

The pain and discomfort associated with periodontal disease can significantly reduce quality of life, making it difficult for individuals to perform daily activities and enjoy social interactions [95].

Additionally, the stress of managing chronic conditions like diabetes and periodontal disease can lead to mental health issues such as anxiety and depression, adding a psychological burden that affects not only the individuals suffering from these conditions but also their families and caregivers. This combination of physical discomfort and psychological stress can deeply impact overall well-being and the ability to maintain a fulfilling lifestyle [96,97].

#### 7.2.4. Social Inequities

Disadvantaged populations, including those with limited access to healthcare and lower socioeconomic status, are disproportionately affected by the dual burden of diabetes and periodontal disease, exacerbating existing health disparities and perpetuating cycles of poverty and poor health. Barriers to accessing comprehensive healthcare services, including dental care, often result in delayed diagnosis and treatment of both conditions, leading to worsening outcomes for affected individuals and communities and further entrenching these disparities [98].

#### 7.2.5. Public Health and Preventive Measures

Public health campaigns to raise awareness about the link between periodontal disease and diabetes are essential, as educating communities about the importance of oral hygiene and regular dental check-ups can help prevent the onset and progression of periodontal disease [99].

Implementing integrated care models that encompass both medical and dental care can significantly improve the management of these conditions, with routine periodontal assessments included in diabetic care protocols and vice versa. Advocacy for policies that promote access to affordable dental and medical care, particularly for high-risk populations, is crucial in reducing the burden of these interconnected diseases. Such policies should also support preventive measures, including community dental programs and regular screenings, to ensure early detection and effective management [30].

## 8. Respiratory Infections

Periodontal pathogens can be aspirated into the lower respiratory tract, leading to respiratory infections, including pneumonia. This risk is particularly significant in elderly individuals and those with chronic respiratory conditions such as chronic obstructive pulmonary disease (COPD) [100]. The oral cavity serves as a reservoir for respiratory pathogens, and poor oral hygiene can exacerbate the risk of respiratory infections [101].

### 8.1. The Pathophysiological Link Between Periodontitis and Respiratory Infections

#### 8.1.1. Aspiration of Periodontal Pathogens

**Figure 7:**
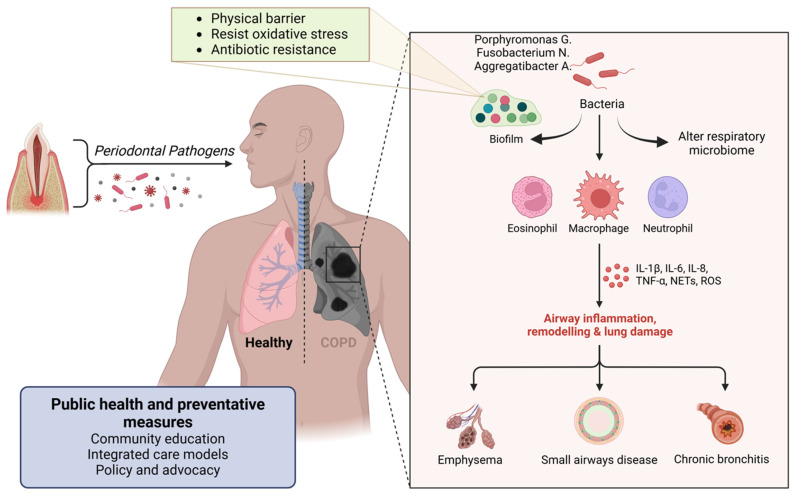
Figure illustrating the connection between periodontal pathogens and respiratory diseases. Oral bacteria, including *Porphyromonas gingivalis*, *Fusobacterium nucleatum*, and *Aggregatibacter* spp., can alter the respiratory microbiome and form biofilms resistant to oxidative stress and antibiotics. These pathogens trigger immune responses involving eosinophils, macrophages, and neutrophils, leading to airway inflammation, lung remodeling, and tissue damage. The resulting conditions include emphysema, small airway disease, and chronic bronchitis, contributing to chronic obstructive pulmonary disease (COPD). Public health and preventive measures, such as community education, integrated care, and policy advocacy, are essential in mitigating these risks. Created with BioRender.com (accessed on 5 March 2025).

Periodontal disease increases the bacterial load in the oral cavity, including pathogens such as *Porphyromonas gingivalis*, *Fusobacterium nucleatum*, and *Aggregatibacter actinomycetemcomitans*. These bacteria can be aspirated into the lower respiratory tract during inhalation [102] (Figure 7).

Aspiration of these pathogens can lead to the development of respiratory infections, including bacterial pneumonia, particularly in vulnerable populations such as the elderly, those with chronic obstructive pulmonary disease (COPD), and individuals with weakened immune systems [103].

#### 8.1.2. Inflammatory Response and Immune System Modulation

The chronic inflammation associated with periodontal disease results in the release of pro-inflammatory cytokines such as interleukin-1 (IL-1), interleukin-6 (IL-6), and tumor necrosis factor-alpha (TNF-α) [1] (Figure 7).

These cytokines can enter the systemic circulation and reach the lungs. Inflammatory mediators can modulate the immune response in the lungs, making them more susceptible to infection. This altered immune environment can impair the clearance of inhaled pathogens and increase the risk of respiratory infections [104].

#### 8.1.3. Bacterial Translocation

Periodontal pathogens can enter the bloodstream through inflamed periodontal tissues and subsequently disseminate to distant sites, including the lungs. This translocation can directly contribute to the development of respiratory infections [103].

Periodontal pathogens such as *Porphyromonas gingivalis*, *Fusobacterium nucleatum*, and *Aggregatibacter actinomycetemcomitans* can adhere to respiratory surfaces, initiating biofilm formation. This process begins with the bacteria’s specific adhesins binding to components of the host tissue. Once adhered to, the bacteria produce extracellular polymeric substances (EPS) composed of polysaccharides, proteins, and DNA, creating a protective matrix that allows the bacterial community to thrive [105]. The biofilm structure acts as a physical barrier, limiting the penetration of immune cells like neutrophils and macrophages, and the bacteria within the biofilm often express different genes that help resist oxidative stress and antimicrobial peptides, further aiding in immune evasion [106] (Figure 7). Antimicrobial treatments face challenges in penetrating the dense biofilm, leading to sublethal concentrations reaching the bacteria, which may allow them to survive and potentially develop resistance [106]. Additionally, within biofilms, a subset of bacteria may enter a dormant state as persister cells, which are highly resistant to antibiotics and can repopulate the biofilm once treatment is discontinued [107].

The proximity of bacterial cells also facilitates the exchange of genetic material, including antibiotic resistance genes. This persistence of biofilm-associated infections can lead to chronic respiratory infections, such as pneumonia, particularly in individuals with compromised immune systems, and increases morbidity due to the difficulty in eradicating these infections with standard antimicrobial treatments [107].

#### 8.1.4. Oral and Respiratory Microbiome Interaction

Periodontal disease, characterized by chronic inflammation and infection of the gums, often leads to dysbiosis, a disruption in the balance of the oral microbiome. In a healthy state, the oral microbiome is composed of a diverse community of bacteria that coexist in harmony [108].

However, periodontal disease shifts this balance, favoring the overgrowth of pathogenic bacteria such as *Porphyromonas gingivalis*, *Tannerella forsythia*, and *Treponema denticola*, among others [109]. This dysbiotic state results in a reduction in beneficial bacteria and an increase in the virulence and metabolic activity of pathogenic species, which exacerbate inflammation and tissue destruction in the oral cavity [110].

When these pathogenic oral bacteria are aspirated into the lungs, they can influence the composition and balance of the respiratory microbiome. Normally, the respiratory tract has a microbial community that helps to protect against the colonization of harmful pathogens by occupying ecological niches and maintaining immune homeostasis [111].

However, the introduction of dysbiotic oral bacteria can disrupt this balance, leading to a shift in the respiratory microbiome. This disruption can weaken the natural defenses of the respiratory tract, creating a more conducive environment for the colonization and growth of opportunistic pathogens [112].

As the respiratory microbiome becomes altered, pathogenic bacteria such as *Streptococcus pneumoniae*, *Pseudomonas aeruginosa*, and *Klebsiella pneumoniae* may find it easier to establish themselves in the lungs. This increases the risk of respiratory infections, including pneumonia, chronic obstructive pulmonary disease (COPD) exacerbations, and even ventilator-associated pneumonia (VAP) in hospitalized patients [113].

The altered respiratory microbiome may also contribute to chronic inflammation in the lungs, further compromising respiratory health. This connection between oral and respiratory health underscores the importance of maintaining good oral hygiene and managing periodontal disease, not only to preserve oral health but also to reduce the risk of respiratory complications [111].

### 8.2. The Broader Impact of Periodontal Disease on the Respiratory System and Society

#### 8.2.1. Increased Healthcare Costs

The treatment of respiratory infections, particularly in vulnerable populations, incurs significant healthcare costs, encompassing expenses for hospitalization, antibiotic therapy, and intensive care in severe cases. This financial strain extends beyond individual patients, contributing to a broader economic burden on healthcare systems, especially in communities with limited resources. The cost of managing respiratory infections related to periodontal disease can be substantial, straining public health budgets and exacerbating existing disparities in access to healthcare [114].

#### 8.2.2. Increased Morbidity and Mortality

Respiratory infections pose a significant risk to vulnerable populations, including the elderly, individuals with chronic diseases such as COPD and diabetes, and immunocompromised patients, who are more likely to experience severe outcomes and complications [115].

The impact of these infections extends beyond individual health, contributing to increased morbidity and mortality within the community, which in turn reduces overall life expectancy and quality of life. The heightened susceptibility of these at-risk groups underscores the importance of targeted preventive measures to protect community health and mitigate the broader impact of respiratory infections [116]. 

#### 8.2.3. Productivity Loss

Individuals suffering from respiratory infections often experience work absenteeism, leading to lost productivity that impacts not only the affected individuals but also the broader economy. This absenteeism can disrupt workplace efficiency and contribute to economic losses on a larger scale [115].

Additionally, recurrent respiratory infections can develop into chronic respiratory conditions, further diminishing an individual’s ability to work and actively participate in community activities. The compounded effect of frequent absenteeism and the progression to chronic health issues can significantly impair an individual’s quality of life and economic stability, underscoring the importance of effective prevention and management strategies for respiratory infections [117].

#### 8.2.4. Quality of Life and Mental Health

Respiratory infections can lead to significant physical discomfort, manifesting as symptoms like coughing, shortness of breath, and fatigue, which interfere with daily activities and reduce overall quality of life [116]. This physical burden is often accompanied by psychological stress, as the ongoing struggle with recurrent infections and the potential development of chronic health conditions can induce anxiety and depression. These mental health challenges not only affect the well-being of the individuals suffering from the infections but also impact their families, creating a ripple effect that extends beyond the physical symptoms to influence emotional and psychological health [118].

### 8.3. Public Health and Preventive Measures

Community Education: Public health campaigns to raise awareness about the link between oral health and respiratory infections are essential. Educating communities about the importance of oral hygiene and regular dental check-ups can help prevent periodontal disease and subsequent respiratory infections [30].

Integrated Care Models: Implementing integrated healthcare models that include dental care as part of routine medical assessments can improve the early detection and management of periodontal disease, reducing the risk of respiratory infections [119].

Policy and Advocacy: Advocating for policies that promote access to comprehensive dental and medical care, particularly for high-risk populations, can reduce the burden of respiratory infections. Policies should support preventive measures such as community dental programs, vaccination, and smoking cessation initiatives [120].

## 9. Adverse Pregnancy Outcomes

Pregnant women with periodontal disease are at an increased risk of adverse pregnancy outcomes, such as preterm birth, low birth weight, and pre-eclampsia. The inflammatory mediators released during periodontitis, including prostaglandins and tumor necrosis factor-α, can induce labor prematurely [121].

### 9.1. The Pathophysiological Link Between Periodontitis and Adverse Pregnancy Outcomes

Periodontal disease, particularly periodontitis, involves chronic inflammation of the supporting structures of the teeth due to bacterial infection. This inflammation triggers systemic inflammatory responses that can significantly impact pregnancy outcomes [122].

The detailed mechanisms through which periodontal disease influences adverse pregnancy outcomes include the following.

### 9.2. Microbial Pathway

Periodontal pathogens such as *Porphyromonas gingivalis*, *Tannerella forsythia*, and *Treponema denticola* can enter the bloodstream through inflamed periodontal tissues.

These bacteria can reach the placental tissues, directly infecting the placenta and amniotic fluid, potentially leading to adverse pregnancy outcomes such as preterm birth and low birth weight [123].

### 9.3. Inflammatory Pathway

In response to periodontal infection, the body releases pro-inflammatory cytokines such as interleukin-6 (IL-6), tumor necrosis factor-alpha (TNF-α), and prostaglandin E2 (PGE2) [124].

**Figure 8:**
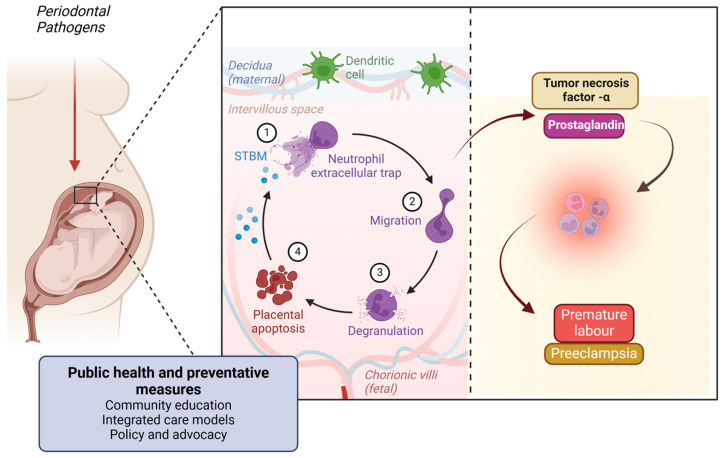
Figure illustrating the link between periodontal pathogens and adverse pregnancy outcomes. Periodontal bacteria can reach the placenta, triggering an immune response involving neutrophil extracellular traps, migration, and degranulation. This leads to placental apoptosis and inflammation, mediated by tumor necrosis factor-α (TNF-α) and prostaglandins. These inflammatory mediators contribute to pregnancy complications such as premature labor and pre-eclampsia. Public health and preventive measures, including community education, integrated care, and policy advocacy, are essential in mitigating these risks. Created with BioRender.com (accessed on 5 March 2025).

These cytokines can enter the bloodstream and reach the uterine environment, causing inflammation of the uterine tissues. Elevated levels of PGE2 and other inflammatory mediators can stimulate uterine contractions, leading to premature labor. Chronic systemic inflammation can also disrupt the normal endocrine and immune functions required for maintaining pregnancy, contributing to complications like pre-eclampsia [125] (Figure 8).

### 9.4. Immune Response Pathway

The maternal immune system reacts to periodontal pathogens by producing antibodies and other immune mediators. This immune response can inadvertently target placental tissues, causing inflammation and damage that can compromise fetal development. Maternal periodontal disease can also alter the maternal-fetal immune balance, affecting the placenta’s ability to support the developing fetus [126].

## 10. The Broader Impact of Periodontal Disease on Pregnancy Outcomes and Society

### 10.1. Community Impact

The impact of adverse pregnancy outcomes due to periodontal disease extends beyond individual health, affecting community health and socioeconomic stability.

Increased rates of preterm birth and low birth weight associated with maternal periodontal disease can lead to long-term health issues for affected children, including developmental delays, respiratory problems, and increased susceptibility to chronic diseases [127].

Communities with a high prevalence of periodontal disease may experience higher infant mortality rates and lower overall community health standards. The economic burden of managing adverse pregnancy outcomes is substantial [128].

Preterm births and low birth-weight infants often require extended hospital stays, specialized medical care, and long-term health interventions [129].

Among adverse pregnancy outcomes, pre-eclampsia and eclampsia warrant particular emphasis due to their substantial contribution to maternal mortality worldwide, especially in rural and underserved areas [121,124]. These hypertensive disorders are often fatal if undetected or untreated and are increasingly recognized as inflammatory-mediated conditions [121]. Evidence suggests that periodontal disease may exacerbate systemic inflammation and endothelial dysfunction, potentially triggering the vascular anomalies seen in pre-eclampsia [121,125]. Elevated levels of circulating cytokines and microbial by-products from periodontal tissues could influence placental function, immune tolerance, and angiogenesis [122]. Given that maternal deaths due to pre-eclampsia and eclampsia are often more frequent than those from preterm birth in low-resource settings, early periodontal screening and intervention may represent a valuable strategy in maternal health care, especially for high-risk populations [125,126,127,128].

### 10.2. Socioeconomic Consequences

Adverse pregnancy outcomes can have far-reaching socioeconomic consequences, affecting not just individual families but entire communities. Beyond the immediate emotional and psychological distress, families may experience chronic stress, depression, and anxiety due to the loss of a child or long-term health complications of the newborn. This psychological burden can disrupt family dynamics, contribute to strained relationships, and diminish overall well-being [130]. Financially, the cost of medical care for infants with health complications—such as preterm birth, low birth weight, or congenital conditions—places a significant financial strain on families, especially in low-resource settings where access to affordable healthcare is limited. Many families incur out-of-pocket expenses for hospitalizations, neonatal intensive care, medications, and ongoing therapy, which can push them further into financial distress and reinforce cycles of poverty [131]. From a societal perspective, reduced productivity and economic losses emerge as parents, particularly mothers, may be forced to take extended leave from work or even exit the workforce entirely to provide full-time care. This issue is particularly detrimental for women in low-income and marginalized communities, where limited maternity protections and workplace policies may lead to job insecurity and income loss [130]. Moreover, health inequities exacerbate these outcomes, disproportionately affecting vulnerable populations, including racial and ethnic minorities, indigenous communities, and those living in rural areas. Geographic disparities and healthcare deserts—areas with inadequate maternal and neonatal healthcare facilities—further limit access to timely and quality care, increasing the likelihood of poor pregnancy outcomes and deepening socioeconomic inequalities [130].

Addressing these consequences requires targeted interventions, such as policy-driven financial support, expanded maternal and child healthcare services, and community-based programs that provide emotional, psychological, and financial assistance to affected families. By reducing healthcare disparities and improving access to quality prenatal and postnatal care, societies can mitigate the long-term socioeconomic burden of adverse pregnancy outcomes [132].

Adverse pregnancy outcomes can affect families’ emotional and psychological well-being, leading to increased stress and potential mental health issues.

The loss of productivity due to caring for infants with health complications can impact family income and contribute to poverty cycles within communities [133].

### 10.3. Educational and Developmental Impact

Children born with complications related to preterm birth and low birth weight may face challenges in cognitive and physical development, affecting their educational achievements and future opportunities. These developmental challenges can perpetuate a cycle of disadvantage and limit the overall growth potential of communities [134].

### 10.4. Public Health Strategies to Mitigate the Impact of Periodontal Disease on Pregnancy Outcomes

To mitigate the impact of periodontal disease on adverse pregnancy outcomes, targeted public health strategies are essential. Oral health education should focus on community-based programs that raise awareness about the importance of oral hygiene during pregnancy while providing resources and training for expectant mothers on proper oral care and routine dental check-ups [27]. Improving access to dental care is crucial, particularly in underserved communities, by integrating dental services into prenatal care programs and ensuring affordable treatment options [27]. Early screening and intervention during prenatal visits should be encouraged, with a focus on collaboration between obstetricians, dentists, and other healthcare providers to manage periodontal health effectively [27]. Policy and advocacy efforts should push for maternal oral health initiatives, securing funding for dental care programs and establishing guidelines for oral health assessments in prenatal care. Additionally, research and innovation should be prioritized to further explore the link between periodontal disease and pregnancy outcomes, leading to advanced preventive and therapeutic strategies [135]. By implementing these comprehensive public health measures, the burden of periodontal disease in pregnancy can be significantly reduced, improving both maternal and fetal health.

## 11. Alzheimer’s Disease

Alzheimer’s disease (AD) is a neurological condition that becomes more prevalent as individuals get older [25]. The start of it might occur either early or late. The hallmarks of AD include prominent inflammatory characteristics, activated microglia, and elevated proinflammatory cytokine levels that exacerbate the inflammatory state of the central nervous system (CNS). Periodontitis is a common oral disease caused by gram-negative anaerobic bacteria [25]. Periodontitis may be classified as a “low-grade systemic disease” due to the release of proinflammatory cytokines into the bloodstream and increased C-reactive protein (CRP) levels. Inflammation is recognized as a crucial factor in the progression of both periodontitis and AD, functioning as a bridge between the two diseases [136].

### 11.1. The Pathophysiological Link Between Periodontitis and Alzheimer’s Disease

#### 11.1.1. Chronic Inflammation and Neuroinflammation

Periodontal disease is characterized by chronic inflammation due to bacterial infection. This leads to the release of pro-inflammatory cytokines such as interleukin-6 (IL-6), tumor necrosis factor-alpha (TNF-α), and C-reactive protein (CRP) into the bloodstream [21,136].

**Figure 9:**
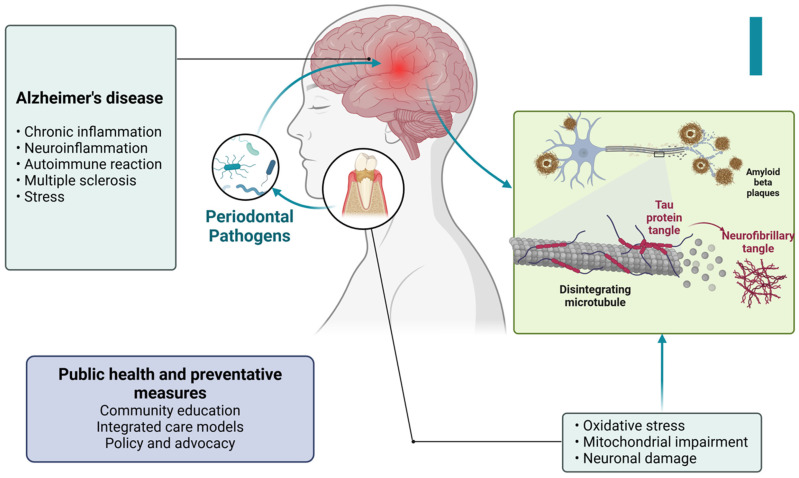
Figure illustrating the connection between periodontal pathogens and Alzheimer’s disease. Periodontal bacteria contribute to chronic inflammation and neuroinflammation, triggering oxidative stress, mitochondrial dysfunction, and neuronal damage. These processes lead to the formation of tau protein tangles and amyloid-beta plaques, which are characteristic of Alzheimer’s pathology. Additionally, factors such as autoimmune reactions, multiple sclerosis, and stress further exacerbate neurodegenerative changes. Public health and preventive measures, including community education, integrated care, and policy advocacy, play a crucial role in mitigating these risks. Created with BioRender.com (accessed on 5 March 2025).

Chronic systemic inflammation can disrupt the integrity of the blood–brain barrier, allowing inflammatory mediators and peripheral immune cells to enter the brain. Once in the brain, these inflammatory mediators contribute to neuroinflammation, a key feature in the pathogenesis of Alzheimer’s disease (AD). Neuroinflammation can promote the accumulation of amyloid-beta (Aβ) plaques and tau protein tangles, which are hallmark features of AD [137] (Figure 9).

#### 11.1.2. Direct Bacterial Invasion

Periodontal pathogens such as *Porphyromonas gingivalis* and their virulence factors (e.g., gingipains) have been detected in the brains of Alzheimer’s patients. These bacteria can reach the brain through the bloodstream or along cranial nerves such as the trigeminal nerve [138].

**Figure 10:**
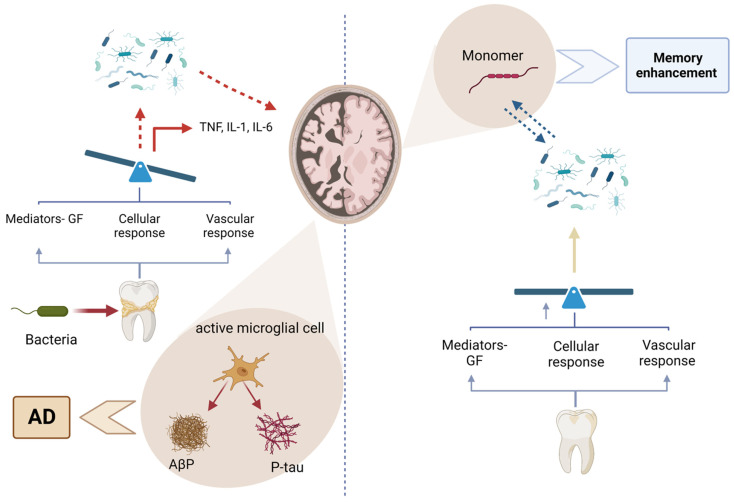
Figure illustrating the relationship between periodontal bacteria and Alzheimer’s disease (AD). On the left, bacterial infections trigger an immune response, activating microglial cells that release proinflammatory cytokines (TNF, IL-1, and IL-6), leading to neuroinflammation and the accumulation of amyloid-beta plaques (Aβ) and phosphorylated tau (P-tau), which contribute to AD progression. On the right, the breakdown of these pathological aggregates into monomers is associated with potential memory enhancement. The balance between inflammatory and protective responses influences disease outcomes, emphasizing the importance of periodontal health in neurodegenerative disease prevention. Created with BioRender.com (accessed on 5 March 2025).

In response to bacterial invasion, neurons may produce amyloid-beta as an antimicrobial peptide. While initially protective, excessive amyloid-beta accumulation leads to plaque formation, contributing to AD pathology [139] (Figure 10).

Bacterial toxins and inflammatory mediators can also promote the hyperphosphorylation of tau proteins, leading to the formation of neurofibrillary tangles, another hallmark of AD [140].

#### 11.1.3. Oxidative Stress

Chronic inflammation in periodontal disease generates reactive oxygen species, leading to oxidative stress. Oxidative stress can damage neuronal cells and exacerbate the pathogenesis of AD by promoting amyloid-beta aggregation and tau hyperphosphorylation. Oxidative stress also impairs mitochondrial function in neurons, leading to energy deficits and further neuronal damage [141].

#### 11.1.4. Immune Response and Autoimmunity

Chronic periodontal infection can lead to prolonged activation of the immune system. Immune cells such as microglia in the brain become overactive, contributing to chronic neuroinflammation [142]. Molecular mimicry between bacterial antigens and neuronal antigens can lead to autoimmune reactions, where the immune system mistakenly attacks brain tissues, contributing to AD progression [58].

### 11.2. The Broader Impact of Periodontal Disease and Alzheimer’s Disease on Society

#### 11.2.1. Increased Prevalence of Alzheimer’s Disease

The link between periodontal disease and Alzheimer’s disease suggests that communities with a high prevalence of periodontal disease may face increased rates of Alzheimer’s, exacerbating the public health challenges associated with an aging population and the rising incidence of neurodegenerative diseases [25].

The management of Alzheimer’s disease demands extensive medical, social, and long-term care services, which significantly escalates healthcare costs and resource allocation. This growing healthcare burden strains healthcare systems, particularly in low- and middle-income countries, where resources are already limited, further complicating efforts to address the needs of a rapidly aging population [143].

#### 11.2.2. Economic Impact

Direct costs associated with Alzheimer’s disease encompass medical care, medications, and long-term care facilities, with expenses escalating as the disease progresses and the need for specialized care intensifies. Indirect costs also contribute significantly to the economic burden, as caregivers often need to take time off work to care for affected family members, leading to lost productivity. This dual financial strain on both families and communities can be substantial, potentially leading to financial hardship and even poverty, particularly as the demands of caregiving and the costs of care continue to rise [144].

#### 11.2.3. Quality of Life and Caregiver Burden

Alzheimer’s disease significantly diminishes the quality of life of patients by impairing cognitive functions, disrupting daily activities, and eroding their independence. As the disease progresses, patients face increasing disability and become heavily reliant on caregivers [145].

This situation places immense emotional, physical, and financial stress on family members and caregivers, often leading to caregiver burnout, depression, and a reduced quality of life for the caregivers themselves. The dual impact of Alzheimer’s disease on both patients and their caregivers underscores the profound challenges posed by this condition [146].

#### 11.2.4. Social and Mental Health Impact

Individuals with Alzheimer’s disease and their families often face social stigmatization, leading to isolation and reduced social support, which can further exacerbate mental health issues like depression and anxiety [147].

The increasing prevalence of Alzheimer’s disease underscores the need for community resources such as support groups, respite care services, and educational programs for caregivers and families. These resources are vital for mitigating the social isolation and mental health challenges associated with the disease, providing essential support to both patients and their caregivers [148].

### 11.3. Public Health and Preventive Measures

Community Education: Public health campaigns to raise awareness about the link between oral health and Alzheimer’s disease are crucial. Educating communities about the importance of maintaining good oral hygiene and regular dental visits can help reduce the prevalence of periodontal disease and its potential impact on AD [149].

Integrated Care Models: Developing integrated healthcare models that include routine oral health assessments as part of regular medical check-ups, especially for older adults, can aid in the early detection and management of periodontal disease, potentially reducing the risk of AD [150].

Policy and Advocacy: Advocating for policies that promote access to comprehensive dental care, particularly for elderly populations, can help mitigate the impact of periodontal disease on Alzheimer’s disease. Policies should also support research into the links between oral health and neurodegenerative diseases, promoting preventive measures and innovative treatments [151].

## 12. Impact of Periodontal Disease on Quality of Life and Self-Esteem in Young Individuals

### 12.1. Quality of Life and Self-Esteem in Young Individuals

Periodontal disease can significantly impact the quality of life and self-esteem of young individuals. The visible symptoms of the disease, such as gum inflammation, bleeding, and bad breath, can lead to embarrassment and social anxiety. For young people, who are often particularly conscious of their appearance and social acceptance, these symptoms can result in a profound psychological impact [152]. Adolescents and young adults may withdraw from social interactions to avoid the stigma associated with poor oral hygiene and the visible signs of periodontal disease. This social withdrawal can lead to feelings of isolation and loneliness, further exacerbating mental health issues such as depression and anxiety [153]. The condition of one’s teeth and gums is closely linked to self-esteem. Young individuals with periodontal disease may experience diminished self-confidence, affecting their willingness to engage in social and professional activities. This can have long-term consequences on their personal and professional development [154]. Poor oral health can also affect academic performance and job prospects. Young individuals may feel self-conscious during interviews or presentations, hindering their ability to perform well in educational and career settings [155].

### 12.2. Psychological and Social Effects

Self-Image and Confidence: The loss of teeth and the visible signs of periodontal disease can affect an older adult’s self-image and confidence. This can lead to reduced participation in social activities and a decrease in overall life satisfaction [156].

Independence and Quality of Life: The functional impairments caused by periodontal disease can affect an older adult’s ability to live independently. Difficulty in eating and maintaining proper nutrition can lead to a decline in physical and mental health, necessitating additional care and support [157].

### 12.3. Reduced Chewing Capacity in Older Adults

For older adults, periodontal disease can lead to significant functional impairments, particularly in chewing and overall oral function. The progression of the disease often results in the loss of teeth and damage to the supporting structures, leading to reduced chewing efficiency and compromised nutrition [158]. Difficulty in chewing can cause older adults to avoid certain types of food, particularly those that are hard or fibrous, such as fruits, vegetables, and meats. This selective eating can result in nutritional deficiencies, further compromising their overall health and well-being. Inefficient chewing can also lead to larger food particles entering the digestive tract, which can cause digestive discomfort and impair the absorption of nutrients [159]. This can exacerbate existing health conditions and reduce overall quality of life. Moreover, the inability to maintain a balanced diet due to periodontal disease can lead to weight loss, weakened immune function, and increased vulnerability to infections and chronic illnesses. This decline in health can be particularly detrimental for older adults, who may already be managing multiple health conditions [160,161] (Figure 6). Given these significant impacts on different age groups, it is evident that periodontal disease affects individuals across their lifespan in profound ways. Addressing these issues requires a comprehensive approach that includes preventive care, early detection, and effective treatment strategies tailored to the specific needs of different age groups. Public health interventions must prioritize education on oral hygiene, access to affordable dental care, and integrated healthcare solutions that consider the systemic effects of periodontal disease [30].

### 12.4. Effective Strategies for Public Health to Address Periodontal Disease

Periodontal disease is a major public health concern that affects millions globally and, if left untreated, can lead to serious health complications. Tackling this issue requires a comprehensive approach that integrates various public health strategies [162] Table 3. As a complex condition, periodontal disease is deeply influenced by social, economic, and policy-related factors, highlighting the need for targeted interventions to address both its causes and consequences effectively.

The increasing awareness of its connection to systemic diseases like cardiovascular disease, diabetes, and adverse pregnancy outcomes highlights the urgent need for a comprehensive, multidisciplinary approach to prevention and management. Public health strategies must emphasize awareness campaigns, community-based interventions, and better access to preventive and therapeutic care to ensure that individuals receive timely diagnosis and treatment [163]. Educational initiatives—ranging from school-based oral health programs to mass media campaigns—are crucial in promoting lifelong oral hygiene habits and reducing modifiable risk factors like tobacco use and excessive sugar consumption [164]. Additionally, expanding access to periodontal care through free or subsidized dental screenings, mobile clinics, and teledentistry can help bridge gaps in oral healthcare, especially for underserved and vulnerable populations [165]. A holistic integration of oral health into general healthcare services is also essential, as it allows medical professionals to screen for periodontal disease, educate patients about its systemic implications, and facilitate early intervention through referrals and shared electronic health records (EHRs) [166,167,168]. At the policy level, legislation promoting tobacco control, reducing sugar in processed foods, and providing workplace oral health benefits can create an environment that fosters better periodontal health outcomes [169]. Moreover, ensuring equitable access to periodontal treatment through Medicaid and Medicare expansions can greatly diminish disparities, particularly among older adults, low-income families, and those in rural communities [170]. The role of research and surveillance is equally critical, as ongoing epidemiological studies and data collection initiatives allow public health authorities to monitor disease trends, evaluate intervention effectiveness, and develop targeted policies. Investing in workforce development, including continuing education for dental professionals, public health training in dental curricula, and expanded roles for community health workers, will help strengthen the healthcare system’s capacity to address periodontal disease at both the individual and population levels [28,171]. Ultimately, tackling periodontal disease requires a coordinated effort involving policymakers, healthcare professionals, researchers, and the public [172,173]. By implementing evidence-based strategies, strengthening preventive care, promoting interdisciplinary collaboration, and addressing key social determinants, public health authorities can significantly reduce the burden of periodontal disease, improve overall oral health, and contribute to better systemic health outcomes worldwide. A future where periodontal disease is effectively managed and prevented is within reach, but achieving this goal will require sustained commitment, innovation, and a proactive, community-driven approach to oral health policy and practice [174,175].

## 13. Conclusions

Periodontal disease is a major global health concern that extends far beyond the oral cavity, contributing to significant systemic morbidity and socioeconomic burden. This narrative review highlights consistent and biologically plausible associations between periodontitis and a range of systemic conditions, including cardiovascular disease, diabetes mellitus, adverse pregnancy outcomes, respiratory infections, and neurodegenerative disorders. These links are primarily mediated by chronic inflammation, microbial translocation, and immune dysregulation, underscoring periodontitis as a modifiable risk factor within the broader context of noncommunicable diseases.

Despite mounting evidence, periodontal disease remains inadequately addressed in public health agendas, with persistent disparities in access to care and a lack of standardized global surveillance systems. These limitations are especially pronounced in low-resource settings, where the disease burden is greatest.

To respond effectively, policymakers should integrate oral health into national noncommunicable disease strategies and primary healthcare frameworks, ensuring equitable access to preventive and therapeutic services. Healthcare professionals must adopt interdisciplinary care approaches that incorporate periodontal screening into routine medical evaluations, particularly for high-risk groups. For patients and communities, targeted public health campaigns and community-based interventions are essential to improve oral hygiene practices and early detection.

Future research should prioritize longitudinal and interventional studies to clarify causal pathways between periodontal and systemic conditions, as well as assess the impact of integrated care models. Innovative approaches—such as host modulation therapies, personalized treatment plans, and digital health solutions—warrant exploration to enhance both prevention and disease management. Addressing these gaps is critical to reducing the global burden of periodontal disease and achieving broader goals of health equity and systemic disease prevention.

## Figures and Tables

**Table 1:** Global prevalence of periodontal disease by region.

Region	Prevalence of Severe Periodontitis (%)	Contributing Factors
Global	11.2%	Smoking, diabetes, and the aging population
Low- and Middle-Income Countries	Higher than the global average	Limited access to dental care, poor oral hygiene, and lack of education [27,28]
High-Income Countries	Significant	Smoking, diabetes, and the aging population [27,28]

**Table 2:** Direct and indirect costs of periodontal disease.

Cost Type	Examples	Impact
Direct Costs	Dental treatments (cleanings, surgeries, and maintenance)	High out-of-pocket expenses and increased healthcare spending
Indirect Costs	Lost productivity due to absenteeism and tooth loss [32,33]	Decreased employability and entrenchment of poverty [32,33]

**Table 3:** Recommended public health strategies to combat periodontal disease.

Strategy	Key Actions	Expected Outcome
Public Awareness Campaigns	Use of mass media, educational programs, and community outreach	Increased awareness, early detection, and prevention
Access to Preventive Services	Free/subsidized screenings and integration with general healthcare [162]	Reduced prevalence and early treatment
Policy and Advocacy	Tobacco control, nutrition policies, and workplace wellness programs [163]	Address root causes and promote healthy behaviors
Research and Data Collection	Epidemiological studies, surveillance systems, and research funding [155]	Better understanding and improved treatment methods
